# Curcumin Blocks Small Cell Lung Cancer Cells Migration, Invasion, Angiogenesis, Cell Cycle and Neoplasia through Janus Kinase-STAT3 Signalling Pathway

**DOI:** 10.1371/journal.pone.0037960

**Published:** 2012-05-25

**Authors:** Cheng-Liang Yang, Yong-Yu Liu, Ye-Gang Ma, Yi-Xue Xue, De-Gui Liu, Yi Ren, Xiao-Bai Liu, Yao Li, Zhen Li

**Affiliations:** 1 Department of Thoracic Surgery, Liaoning Cancer Hospital and Institute, Shenyang, Liaoning Province, China; 2 Department of Neurobiology, College of Basic Medicine, China Medical University, Shenyang, Liaoning Province, China; 3 The 96th Class, 7-year Program, China Medical University, Shenyang, Liaoning Province, China; 4 Department of Neurosurgery, Shengjing Hospital of China Medical University, Shenyang, Liaoning Province, China; Jawaharlal Nehru University, India

## Abstract

Curcumin, the active component of turmeric, has been shown to protect against carcinogenesis and prevent tumor development. However, little is known about its anti-tumor mechanism in small cell lung cancer (SCLC). In this study, we found that curcumin can inhibit SCLC cell proliferation, cell cycle, migration, invasion and angiogenesis through suppression of the STAT3. SCLC cells were treated with curcumin (15 µmol/L) and the results showed that curcumin was effective in inhibiting STAT3 phosphorylation to downregulate of an array of STAT3 downstream targets ,which contributed to suppression of cell proliferation, loss of colony formation, depression of cell migration and invasion. Curcumin also suppressed the expression of proliferative proteins (Survivin, Bcl-X_L_ and Cyclin B1), and invasive proteins (VEGF, MMP-2, MMP-7 and ICAM-1).Knockdown of STAT3 expression by siRNA was able to induce anti-invasive effects in vitro. In contrast, activation of STAT3 upstream of interleukin 6 (IL-6) leads to the increased *cell* proliferation ,*cell* survival, angiogenesis, invasion, migration and tumor growth. Our findings illustrate the biologic significance of IL-6/JAK/STAT3 signaling in SCLC progression and providenovel evidence that the pathway may be a new potential target for therapy of SCLC. It was concluded that curcumin is a potent agent in the inhibition of STAT3 with favorable pharmacological activity,and curcumin may have translational potential as an effective cancer therapeutic or preventive agent for SCLC.

## Introduction

Signal transducer and activator of transcription 3 (STAT3) protein is a member of a family of latent cytoplasmic transcription factors transmitting signals from the cell surface to the nucleus activated by cytokines and growth factors. The bond of cell surface receptors with ligands, such as interleukin-6 (IL-6) or epidermal growth factor (EGFR), induces tyrosine phosphorylation of STAT3 protein by Janus kinase and growth factor receptor tyrosine kinases [1]. The activated dimeric form of phospho- STAT3 translocates to the nucleus and regulates the expression of genes containing STAT3-binding sites in their promoters [2]. The activation of STAT3 protein is rapid and transient in normal cells. STAT3 regulates fundamental biological processes,including cell proliferation, survival, and development. Recently, accumulating evidence indicates that abnormalities in the Janus kinase (JAK)/signal transducer and activator of transcription (STAT) signaling pathway are involved in the oncogenesis of several cancers [3].

Activated STAT3 (nuclear pSTAT3) is expressed in about 55% of NSCLC tumors, as measured by immunohistochemical analyses[4]–[6]. STAT3 activation is observed in the majority of NSCLC cell lines [7], [8]. In contrast to NSCLC, strong pSTAT3 expression was demonstrated in 100% (10/10) of SCLC tumor tissues tested [9]. However, the mechanism by which dysregulated STAT3 signaling contributes to the progression of human small cell lung cancer (SCLC) has not been elucidated. SCLC is known to have a more aggressive biology with rapid growth and early spread, as well as a common association with paraneoplastic syndromes [10]. Of all histologic types of lung cancer, SCLC is the most sensitive to chemotherapy and radiation, but prognosis remains poor, with an overall median survival following treatment of 10 months and a 5-year survival of 5% [11]. Elderly lung cancer patients and those with poor performance status are not treated with chemotherapy because of the high toxicity of multidrug regimens. Discovery of novel agents with less severe side effects is of great necessity. In clinical treatment of cancer patients, many prescription drugs are derived from natural plant species [12].

Curcumin is derived from turmeric (*Curcuma longa*) and is a natural polyphenol. Curcumin has long been used as a food, coloring agent, and traditional medicine. It is safe and nontoxic and has demonstrable antitumor, antiinflammatory, apoptotic, and antioxidant properties [13]. Especially for its anticarcinogenic property, still has been the subject of a great deal of interest. Increasing evidence indicated that curcumin has anticancer effects against different types of human tumor cells, including of ovarian cancer cells, colon cancer cells and astroglioma cells [14], [15]. This anticancer effects of curcumin were identified through interfering with the cell cycle, inducing apoptosis, and inhibiting the invasive potential of cancers. However, the underlying mechanisms of this anticancer effects are still under investigation, especially for its anti-invasive potential in SCLC cancer.We have shown previously that curcumin inhibits SCLC cell growth and induces SCLC cell apoptosis [16]. In the present study, we investigated the molecular mechanisms by which curcumin suppress migration and invasion in SCLC cells. Our aim was to determine the role of JAK/STAT signaling in SCLC progression and test the hypothesis that curcumin could serve as therapeutic targets.

## Results

Our goal in this study was to determine whether curcumin modulates the growth of SCLC cell lines and, if so, to delineate various mechanisms by which it may mediate its effects. We examined the effects of curcumin on JAKs activation, STAT3 activation and STAT3-regulated gene products and cell growth in SCLC (NCI-H446 and NCI-1688 )cells. In a previous study, we identified a higher p-STAT3 expression level in NCI-H446 cell lines compared with other human SCLC cell lines. We selected NCI-H446 cells for this study.

**Figure 1:**
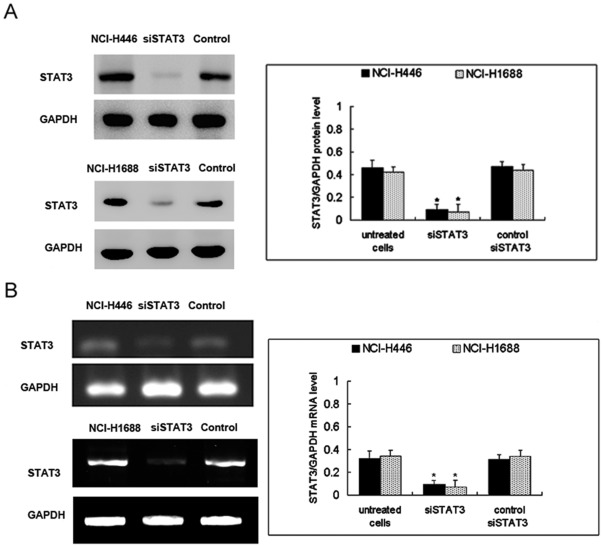
Efficiency of STAT3 siRNA in SCLC cells. Cells were transfected with control siRNA or STAT3 siRNA(si STAT3). After transfection, the expression of STAT3 protein (A) and mRNA (B) was evaluated using Western blot and RT-PCR and compared to untransfected NCI-H446 and NCI-H1688 cells. The columns “NCI-H446 and NCI-H1688 cells” correspond to the untreated cells respectively. The columns “siSTAT3” correspond to the transfected with STAT3 siRNA.The columns “control” correspond to the transfected with control siRNA. Each bar represents the mean ± SD of three independent experiments. *p<0.05, compared with control cells.

IL-6 induces proliferation of human SCLC cells, and curcumin or si STAT3 inhibits it. Curcumin or siSTAT3 inhibits the proliferation of NCI-H446 and NCI-1688 cells. To investigate the role of STAT3 in the functioning of SCLC cells, STAT3 activation and inhibition were induced with IL-6 and with siSTAT3 or curcumin, respectively. After transfection with STAT3 siRNA (siSTAT3) or control siRNA(commercially negative control), the expression of STAT3 was evaluated using Western blot and RT-PCR. We found that siSTAT3 significantly downregulated the protein and mRNA levels of STAT3, compared with control siSTAT3 (Fig. 1).

**Figure 2:**
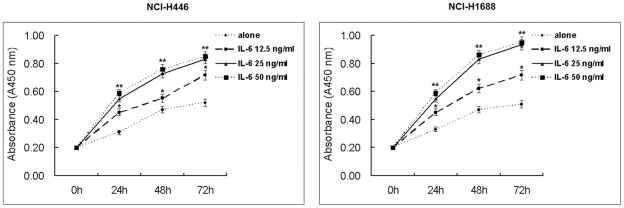
Effect of IL-6 at various concentrations on proliferation of SCLC cells. Cells were treated with IL-6(12.5, 25 or 50 ng/ml) for 24, 48, or 72 h, and cell vitality was estimated using the Brdu assay. Each bar represents the mean ± SD of three independent experiments. *p*<0.05 or ** p<0.01, compared with cells untreated with IL-6.

**Figure 3:**
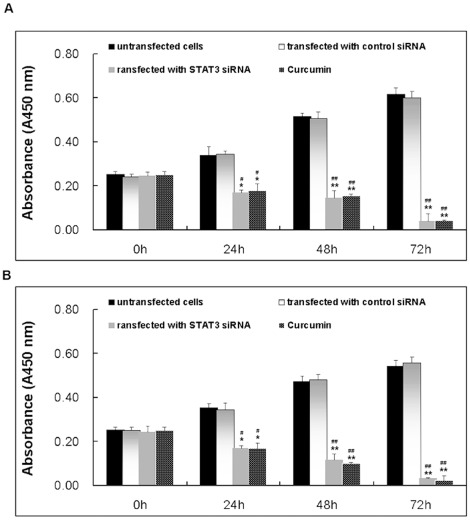
Effect of siSTAT3 or curcumin on proliferation of NCI-H446 cells. Cells were treated with curcumin (15 µM)for 24, 48, or 72 h, and cell vitality was estimated using the Brdu assay. Each bar represents the mean ± SD of three independent experiments. * *p*<0.05 or ** *p*<0.01, compared with the untreated cells. ^#^ *p*<0.05 or^ ##^ *p*<0.01, compared with the transfected with control siRNA cells.

To reconfirm previous reports [17], [18] that IL-6 induces proliferation of cancer cells, we serum-starved NCI-H446 and NCI-1688 cells for 12 h and then cultured them in the absence or presence of different concentrations of IL-6 for 24 h, 48 h and 72 h. IL-6 induced proliferation of NCI-H446 and NCI-1688 in a dose-dependent manner. According to the results of our preliminary experiment, at 25 ng/ml concentrations IL-6 significantly promoted cell proliferation, compared with 12.5 ng/ml concentrations, while there were no significant difference between 25 ng/ml and 50 ng/ml concentrations (Fig. 2). The half maximal inhibitory concentration (IC_50_) value of curcumin was 15 µM in SCLC cells from our previous published paper [16]. Therefore, at 25 ng/ml concentration IL-6 and 15 µM concentration curcumin were used in the following experiment. SiSTAT3 alone or 15 µM curcumin had significant effect on cell proliferation, compared with control cells (Fig. 3). Significant differences were observed between all time points examined, indicating a linear increase in proliferation with increasing exposure times to IL-6 (all *p*<0.05).

**Figure 4:**
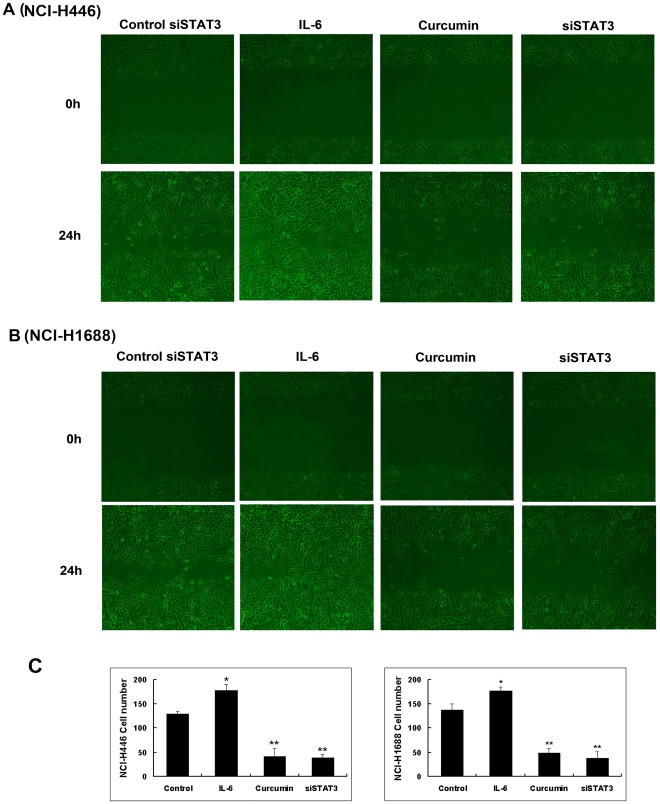
The wound-healing assay for evaluating the inhibitory effects of curcumin on SCLC cell migration. Confluent monolayers of cells were scarred, and repair was monitored microscopically after 24 h of treatment with curcumin 15 µM or IL-6 25 ng/ml. The representative photographs showed the same area at time zero and after 24 h of incubation with or without curcumin. Each bar represents the mean ± SD of three independent experiments. * *p*<0.05 or ** *p*<0.01, compared with control cells.

**Figure 5:**
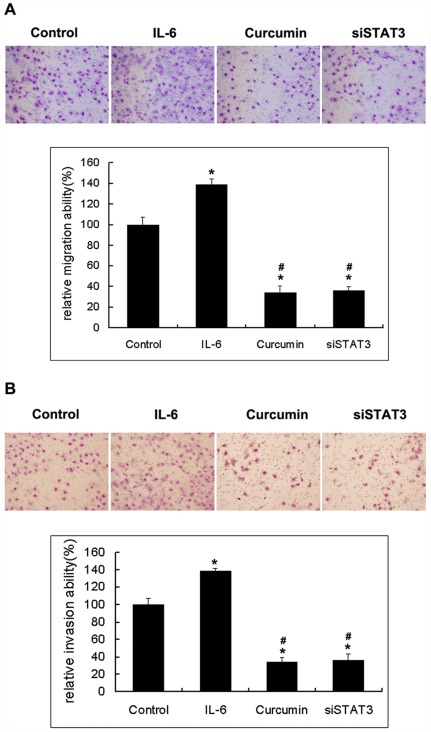
A, the Transwell migration assay showed the inhibitory effects of curcumin on NCI-H446cell migration. After 8 h of incubation with or without the indicated concentration of curcumin, cells that migrate to the lower chamber were fixed, stained, and counted using light microscopy or flurescent microscopy–based high content screening system, as describe in Materials and Methods. Random fields were scanned (four fields per filter of the well) for the presence of cells on the lower side of the membrane. Each bar represents the mean ± SD of three independent experiments. * *p*<0.05, compared with control cells.^ #^ *p*<0.05 compared with cells treated with IL-6 (25 ng/ml). **B,** the effect of curcumin treatment on cell invasion was determined using the Matrigel Invasion Assay System. NCI-H446 cells in serum-free medium with or without curcumin were seeded into the upper chamber of the system. The bottom well was filled with complete medium. After 16 h of incubation, the cells that had invaded through the Matrigel membrane were stained with 20% Giemsa solution and counted under a light microscope (magnification, ×200). The experiments were performed thrice in triplicate. Each bar represents the mean ± SD of three independent experiments. * *p*<0.05, compared with control cells.^ #^ *p*<0.05 compared with cells treated with IL-6 (25 ng/ml).

Curcumin inhibits SCLC cells migration and invasion. The inhibitions of NCI-H446 and NCI-1688 cell migration by curcumin were examined by using wound-healing assays and transwell migration models and results are shown in Fig. 4 and Fig. 5A. Curcumin (15 µmol/L) was able to significantly inhibit NCI-H446 and NCI-1688 SCLC cells migration, and this effect was consistent across both the wound-healing and transwell migration models. In addition, the wound-healing assays and transwell migration model assays indicated that siSTAT3 was able to inhibit cell migration.IL-6 was able to promote cell migration and invasion. The Matrigel-based transwell assay indicated that curcumin or siSTAT3was able to significantly inhibit cell invasion (Fig. 5B). These results clearly suggest that curcumin exhibits anti-migration and anti-invasion effects against SCLC cells.

**Figure 6:**
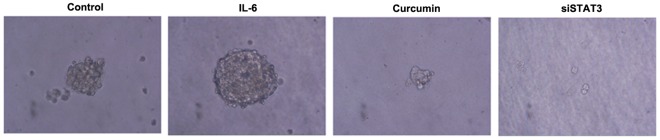
Anchorage-independent growth in soft-agar. Colony formation of the NCI-H446 cells and the indicated stable sublines in soft agar. Cells were observed using an inverted microscope (×400) in soft agar for 15 days. The columns “control” correspond to the transfected with control siRNA.

Importance of the IL-6/JAK/STAT3 pathway in anchorage-independent growth. The anchorage-independent growth of SCLC cells was determined in a soft agar assay [19]. NCI-H446 cells were able to build large aggregates in the soft agar during the 15 days observation period. In contrast, there was no proliferation in the presence of siSTAT3 or curcumin, which suggests a contribution for this pathway in this process. There was a complete promote by the IL-6, suggesting importance of this pathway in anchorage-independent growth (Fig. 6). In the first week, The cells looked quite similar under the microscope except for the fact that the inhibited ones did not proliferate. However, after 7–14 days cells inhibited with curcumin began to change their morphology.

Curcumin causes G_2_/M Phase cell cycle arrest in NCI-H446 cells. To determine whether curcumin inhibits the cell cycle progression of NCI-H446 cells, cells were grown to 70% confluence and the cell cycle distribution was analyzed by flow cytometry after a 12- and 24-h exposure to curcumin (15 µM). It was observed that the percentage of cells in G_2_/M phase with curcumin treatment was 52.2% after 12 h of incubation and decreased to 21.2% after 24 h. The percentage of cells in sub-G_0_/G_1_ phase was 3.5% and 37.1% after 12 and 24 h of incubation, respectively. In control cells the percentage of cells in G_2_/M and Sub-G_0_/G_1_ phase was 21.7% and 1.1% respectively(Table 1).

**Table 1:** Curcumin causes G2/M arrest of SCLC cells.

Group	Sub-G0/G1 phase	G0/G1 phase	S phase	G2/M phase
	Mean ± SD(%)	Mean ± SD(%)	Mean ± SD(%)	Mean ± SD(%)
Control	1.1±2.13	49.2±5.45	27.6±6.81	21.7±5.49
Curcumin 12 h	3.5±1.36	18.7±8.36	24.2±7.12	52.2±4.75*
Curcumin 24 h	37.1±4.29*	28.3±6.47	13.2±6.93	21.2±2.48

Curcumin causes G2/M arrest of NCI-H446 cells. NCI-H446 cells were treated with curcumin (15 µM) for 24 h, and cell cycle distribution was estimated using flow cytometry. Data were present as mean ± SD of three independent experiments. *p<0.05, compared with control cells.

**Figure 7:**
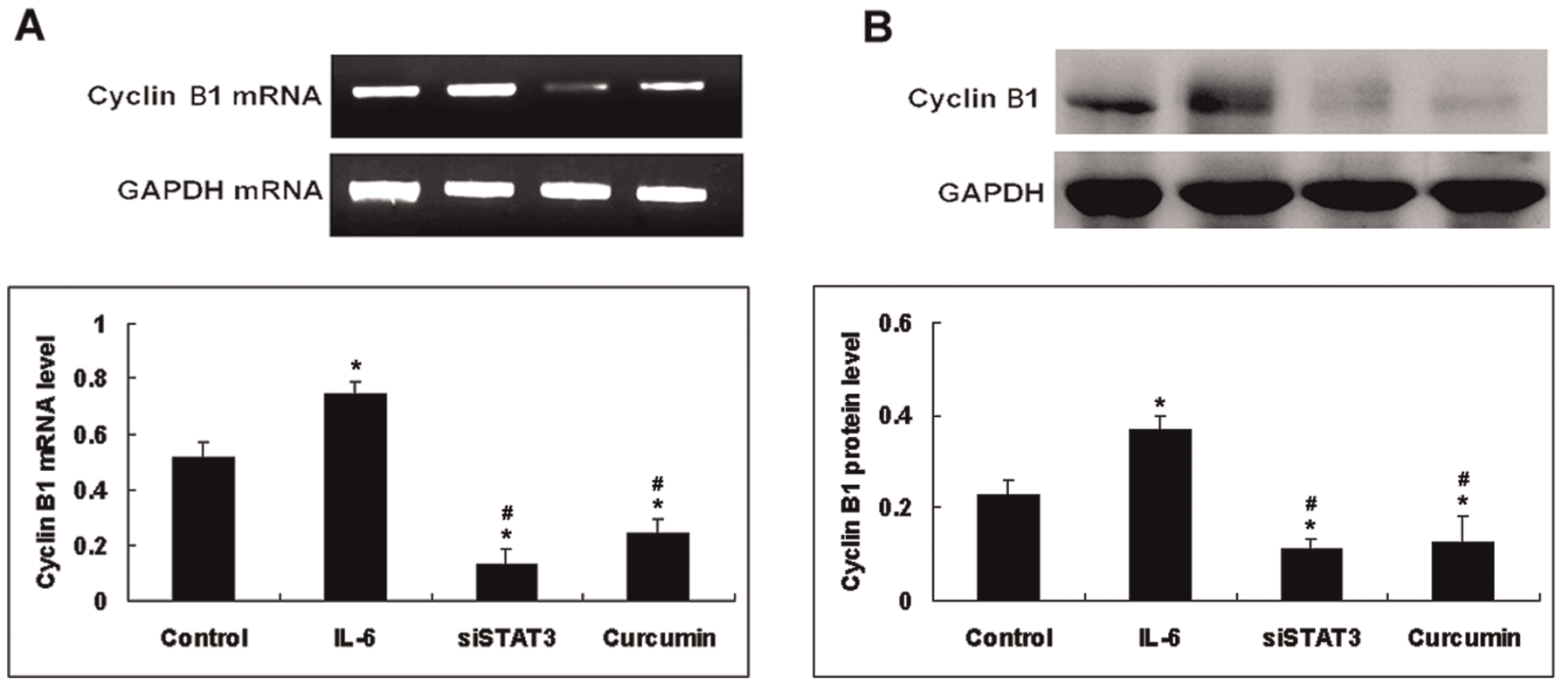
Effect of IL-6, curcumin or siSTAT3 on the expression of CyclinB1. NCI-H446 cells were treated with IL-6 (25 ng/ml)or curcumin (15 µM) for 24 h. The expression levels of these components were estimated using RT-PCR(A) and Western blot(B). Each bar represents the mean ± SD of three independent experiments. * *p*<0.05 compared with control cells.^ #^ *p*<0.05 compared with cells treated with IL-6 (25 ng/ml).

To determine the possible mechanism by which curcumin influences the G_2_/M phase distribution in NCI-H446 cells, the expression of Cyclin B1 was assessed using RT-PCR and Western blot. Compared with control cells, the curcumin significantly downregulated the mRNA and protein levels of Cyclin B1, which is related to G_2_/M phase progression (Fig. 7). The siSTAT3 had significant effect on the levels of Cyclin B1 expression. Compared with control cells, the IL-6 significantly upregulated the protein and mRNA levels of Cyclin B1. Taken together, the results above manifest that curcumin inhibits proliferation of SCLC cancer cell and blocks it in G_2_ phase through suppressing the expression of Cyclin B1.

**Figure 8:**
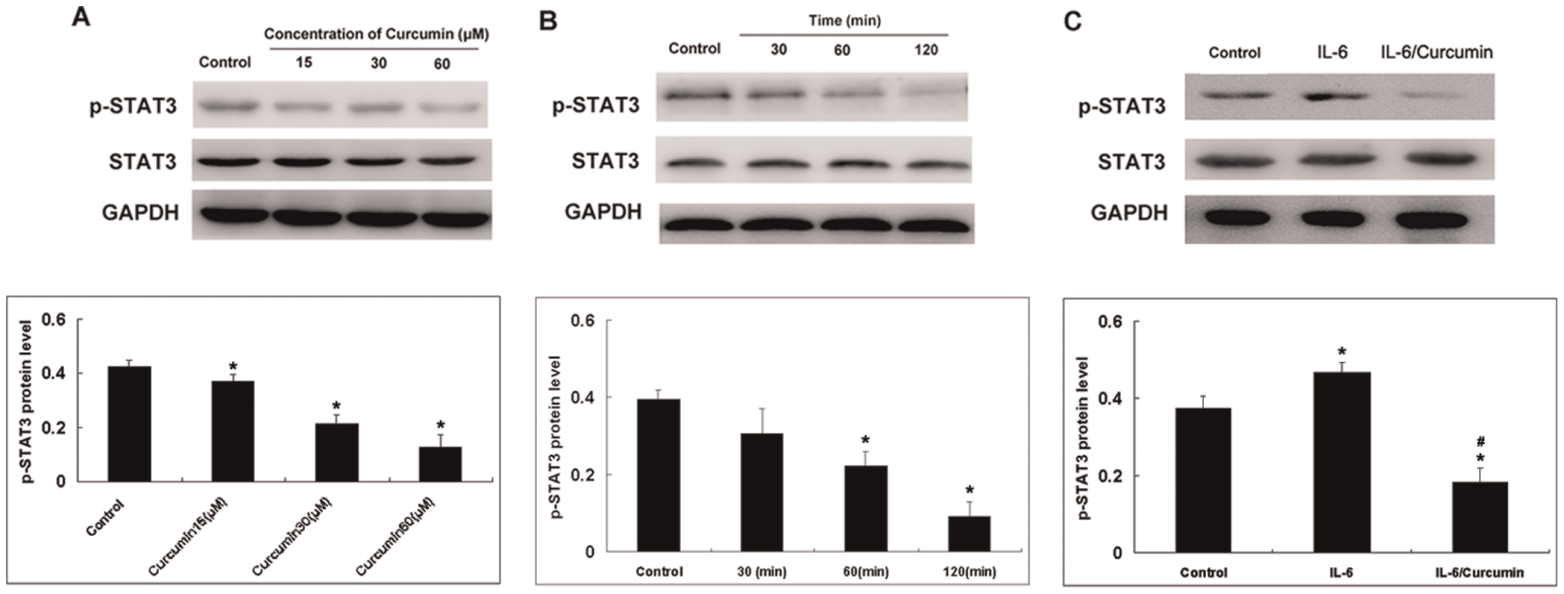
Curcumin attenuates Tyr705p-STAT-3 in SCLC cells. A: To study the dose-dependent effect the NCI-H446cells were treated with different concentrations of curcumin for 1 h. B: To study the time-dependent effect, cells were treated with curcumin (15 µM ) for 30, 60 and 120 min. C: To study the effect of curcumin on IL-6-induced p-STAT-3, cells were cultured asdescribed above except that cells were pretreated with IL-6 (25 ng/ml) for 1 h prior to treatment with curcumin (15 µM) for 1 h. Cells were removed at indicated times and lysed to prepare the whole-cell extract. Cells were harvested and 20 µg of protein from the whole-cell extract was loaded in each lane. GAPDH was used as a loading control. Each bar represents the mean ± SD of three independent experiments. *p<0.05 compared with control cells.^#^ *p*<0.05 compared with cells treated with IL-6 (25 ng/ml).

Curcumin inhibits IL-6 inducible STAT3 phosphorylation in a dose and time dependent manner. To examine if curcumin that was designed to selectively target STAT3 would show similar inhibitory effect on STAT3 phosphorylation, NCI-H446 cells were cultured in serum free medium overnight and then were pre-treated with different concentrations of curcumin for 1 hours or 15 µM curcumin for different time. After the treatment, total protein was extracted and phosphorylated STAT3 and total STAT3 were analyzed by western blot. As shown in Figure 8, curcumin inhibited STAT3 phosphorylation on tyrosine 705 in a dose dependent and time manner. Total STAT3 was not affected by the treatment of curcumin.

Given that IL-6-induced signals are mediated through STAT3 phosphorylation, it is logical to then investigate the effect of IL-6 and curcumin on STAT3 phosphorylation in SCLC cells. Although SCLC cells express high levels of Tyr705p-STAT3 (Fig. 8A and B). Figure 8C shows that IL-6-induced STAT3 phosphorylation in SCLC cells was reduced by curcumin treatment. Exposure of cells to curcumin for 1 h markedly suppressed IL-6-induced STAT3 phosphorylation in NCI-H446 cells.

**Figure 9:**
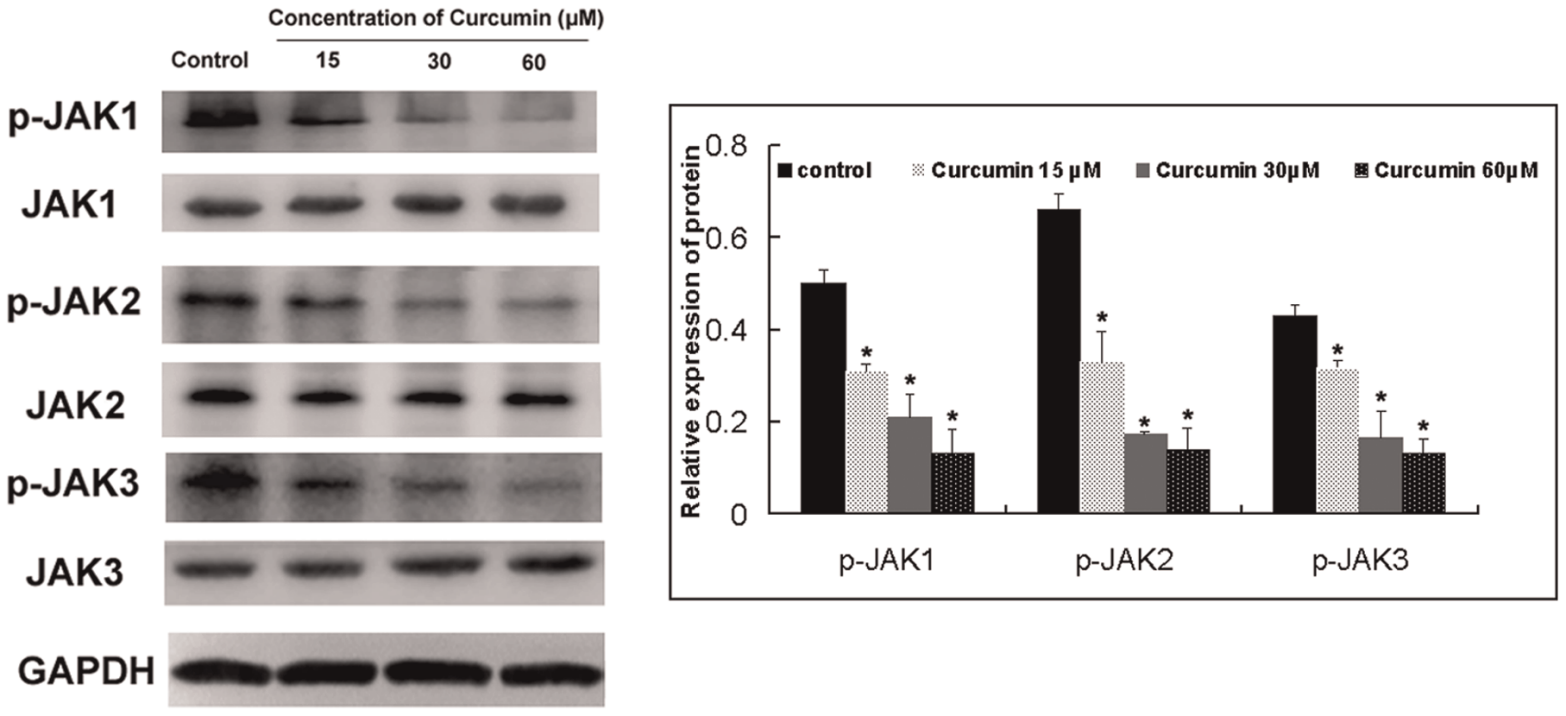
Effect of curcumin treatment on protein expression of P-JAK1, P-JAK2 and P-JAK3. NCI-H446 cells were treated with dfferent concentration of curcumin for 30 min.These proteins were assayed by Western blotting. Equal amounts of total cellular protein (20 µg) were resolved by 10%–15% SDS-PAGE. GAPDH was used as a loading control. Each bar represents the mean ± SD of three independent experiments. * *p*<0.05 compared with control cells.

Curcumin inhibits JAK phosphorylation in SCLC cells. Phosphorylation of STATs depends on the activation of JAKs. Members of the JAK family of protein tyrosine kinases phosphorylate phosphorylate and activate cytoplasmic STAT proteins. JAK-2 is a recognized activator of STAT3 [20]. We investigated whether the inhibitory effects of curcumin on the activation of STAT were due to the suppression of JAK expression in SCLC cells.Curcumin inhibited p-JAK-1, p-JAK-2 and p-JAK-3 expression in NCI-H446 cells (Fig. 9). Expression of total JAK-1, JAK-2 and JAK-3 was not altered by curcumin treatment.

**Figure 10:**
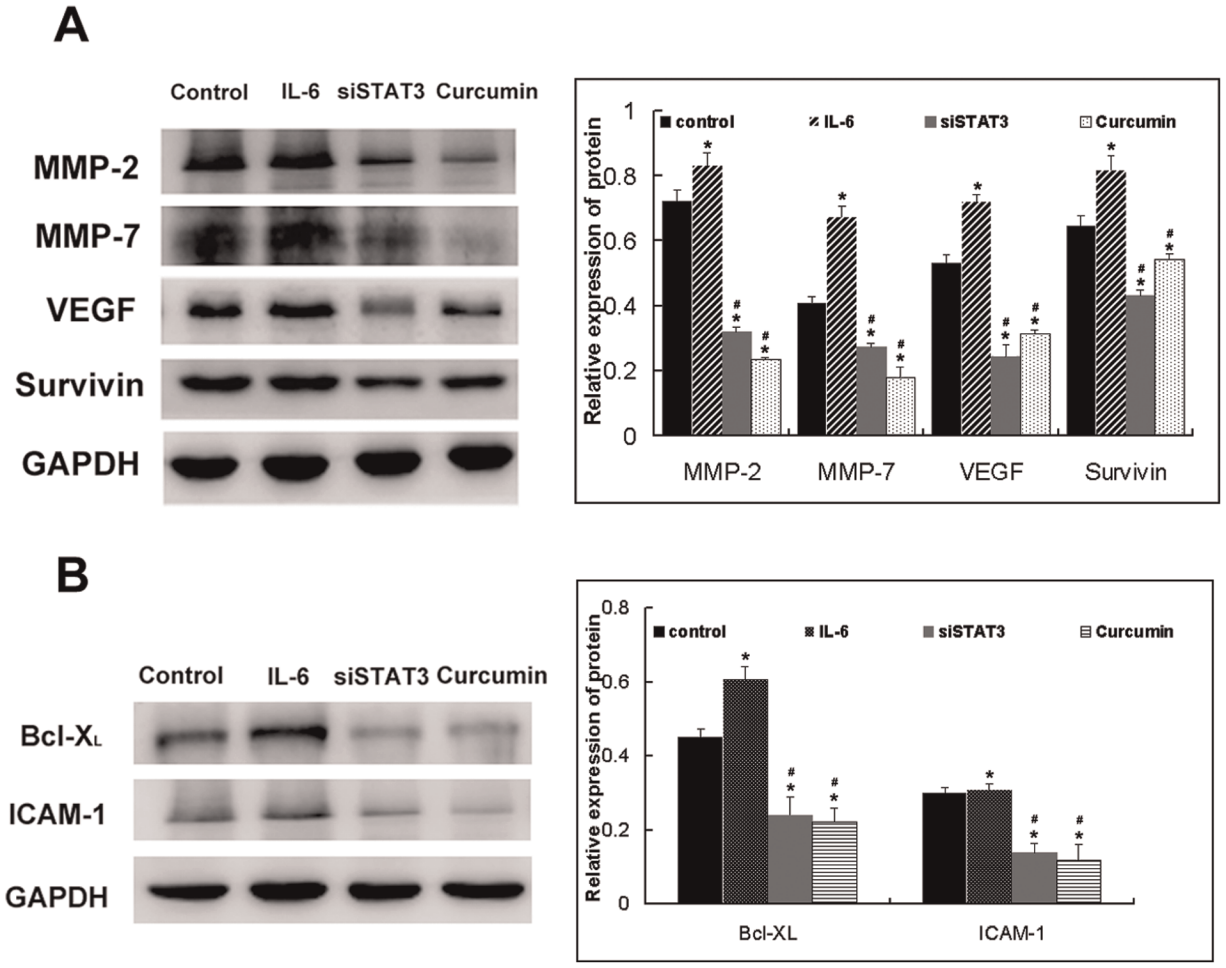
A and B. Effect of curcumin treatment on protein expression of MMP-2, MMP-7, VEGF, Survivin, Bcl-X_L_ and ICAM-1. NCI-H446 cells were treated with 15 µM curcumin for 24 h. These proteins were assayed by Western blotting. Equal amounts of total cellular protein (20 µg) were resolved by 10%–15% SDS-PAGE. GAPDH was used as a loading control. Each bar represents the mean ± SD of three independent experiments. * *p*<0.05 compared with control cells. ^#^ *p*<0.05 compared with cells treated with IL-6(25 ng/ml).

Curcumin-inhibited levels of MMP-2, MMP-7, VEGF, Survivin, Bcl-X_L_ and ICAM-1 in SCLC cells. Levels of migration, invasion and angiogenesis associated proteins in NCI-H446 cells after treatment with curcumin were determined and quantitated by Western blotting. Results were shown in Figure10 indicate that the levels of MMP-2, MMP-7, VEGF and Survivin (Fig 10A). BCL-X_L_ and ICAM-1 (Fig. 10B) were lower than the corresponding control group. These proteins play an important role of cell migration, invasion and angiogenesis. These effects may have led to the inhibition of migration, invasion and angiogenesis from NCI-H446 cells after exposure to curcumin at 15 µM.

## Discussion

SCLC represents a highly malignant and particularly aggressive form of cancer, with early and widespread metastasesand a poor prognosis. To date, there is no data delineating the effects of curcumin on proteins regulating STAT3 signaling in SCLC. Several studies employing genetic and pharmacological approaches to modulate constitutive signal transducer and activator of transcription 3 (STAT3) activity have substantiated the critical role of aberrant STAT3 activity in malignant transformation and tumor progression, and hence endorse STAT3 as a novel cancer drug target [21].

Curcumin (diferuloylmethane), an anti-inflammatory agent used in traditional medicine, has been shown to suppress cellular transformation, proliferation, invasion, angiogenesis, and metastasis through a mechanism not fully understood. Because several genes that mediate these processes are regulated by signal transducer and activator of transcription, we have postulated that curcumin mediates its activity by modulating STAT3 activation. In this study, we investigated the mechanism by which curcumin manifests its effect on STAT3 and STAT3-regulated downstream gene expression. We showed that curcumin down-regulated the expression of STAT3-regulated gene products involved in cellular cycle (Cyclin B1), survival (Bcl-X_L_, Survivin), angiogenesis (vascular endothelial growth factor) and metastasis (matrix metalloproteinase-2, matrix metalloproteinase-7 and intercellular adhesion molecule-1). Traditionally, the cell cycle is segregated into four phases: DNA replication occurs during S phase, and chromosome segregation occurs during M phase. The S and M phases are separated by the so-called gap phases, G_1_ (before DNA replication) and G_2_ (before mitosis). Our study demonstrated that curcumin inhibited the NCI-H446 cells proliferation by inducing G_2_/M cell cycle arrest leading to apoptotic cell death. No significant differences were observed regarding the distribution of cells in the G_0_/G_1_ and S phases. In this study, both protein and mRNA levels of Cyclin B1 were significantly downregulated when cells were treated with curcumin for 24 h. Cyclin B1, which is overexpressed in a variety of tumors, is regulated by STAT3 and is required for cells to advance from the G_2_ phase to the M phase of the cell cycle [22], [23]. This finding demonstrates for the first time that curcumin has an arrest effect on cell cycle progression involving the G_2_/M phase in SCLC cells. It also may explain the antiproliferative effects of curcumin. Our laboratory has shown previously that curcumin can induce SCLC cells apoptosis. In addition to the constitutive activation of STAT3, we were able to demonstrate that stimulation of SCLC cells with IL-6 increased the STAT3 phosphorylation in SCLC cells.Our results strengthen the potential of curcumin as a multitarget drug in anticancer therapy.

Our finding that curcumin inhibited the SCLC cell invasion through down-regulation of the expression of STAT3 regulated gene products involved in cell invasion (e.g., MMP-2 and MMP-7). The down-regulation of VEGF as shown here could explain the antimetastatic activities of curcumin [24]. ICAM-1 has been implicated in carcinogenic processes, and its overexpression by malignant cells has been shown to enhance cellular invasion, induce angiogenesis, regulate antiapoptotic cellular defenses, and augment immunologic resistance through production of prostaglandin E2 [25]. MMP-2 and MMP-9 plays a crucial role in tumor invasion and angiogenesis by mediating degradation of the extracellular matrix, and inhibition of MMP activity has been shown to suppress lung cancer metastasis [26].

To investigate the mechanism of curcumin-induced STAT3 inhibitory effects in SCLC cells, we analyzed proteins upstream of STAT3. The roles of JAK have been implicated in STAT3 activation. As shown in Figure 8C and 9B, phosphorylation of JAK-1, JAK-2 and JAK-3 were suppressed by treatment with curcumin in SCLC cells. It is likely that the inhibition of Tyr705 STAT3 is due to the inhibition of JAK because the inhibition of JAK phosphorylation occurred within 30 min after the treatment, whereas STAT3 phosphorylation was inhibited within 1 hour after the treatment. This suggests that curcumin could manifest its effect on STAT3 activation through the inhibition of JAKs. In a previous study, our laboratory found that curcumin selectively inhibits STAT3 tyrosine phosphorylation but does not affect serine phosphorylation of STAT3 in SCLC cells. These results are in accordance with studies by Saydmohammed et al [27]. They found that curcumin failed to inhibit the phosphorylation of STAT3 at Ser727 in endometrial and ovarian cancer cells. Upon IL-6 stimulation, STAT3 is quickly phosphorylated on Tyr705 and translocated to the nucleus. Our results further indicate that IL-6 mediated STAT3 Tyr705 phosphorylation is mainly a nuclear event.

In many tumors and cancer cells, constitutive activation of STAT3 is IL-6 dependent, through either autocrine or paracrine signaling, which seems to be widely expressed in cancer cell lines [28]. In some cases, the activation of the STAT3 pathway is correlated with brisk intratumoral inflammatory cells infiltration, which means that STAT3 activation may be via paracrine mechanisms [29]. As to this case, inhibition of STAT3 caused decreased production of IL-6.The activation of STAT3 pathway has been studied in patients’ tumor tissue samples. The levels of p-STAT3 expression correlated with the survival rate and severity of chordoma [30]. Recent studies have also linked the activation of STAT3 pathway to high histologic grade and advanced stage in several cancers [31]. These results suggested that STAT3 may not only serve as a predictive marker of drug resistance, but also as a prognosis marker of tumors [32].

In summary, prior to this report, there was no information on the status of JAK/STAT3 signaling proteins in SCLC cells and their regulation by curcumin. This study is the first to have examined in detail curcumin anti-cancer mechanistic role of JAK/STAT3 signaling in SCLC tumorigenesis and progression. The significance of this study is that we have identified a perturbance in a plethora of JAK/STAT signaling proteins in cancer cells. Thus, STAT3 plays a significant role in SCLC oncogenesis and could be a potential therapeutic target for SCLC treatment.

## Materials and Methods

### Chemicals and Antibodies

Curcumin (1,7-bis[4-hy-droxy-3-methoxyphenyl]-1,6-heptadiene-3,5-dione) was purchased from Sigma-Aldrich(St. Louis, MO), dissolved in DMSO at a stock concentration of 10 mM, and diluted to the indicated concentration with RPMI 1640 medium. The antibodies for Survivin, Bcl-X_L_, Cyclin B1, P-JAK1, JAK1, P-JAK2, JAK2, P-JAK3, JAK3, Tyr^705^-pSTAT3 and STAT3 were purchased from Cell Signaling Technology (Beverly,MA). The antibodies for VEGF, ICAM-1, MMP-2, MMP-7, and GAPDH were purchased from Santa Cruz Biotechnology (Santa Cruz, CA, USA). siSTAT3 and Lipofectamine 2000 were purchased from Invitrogen (Carlsbad, CA, USA). Other chemicals were purchased from Sigma-Aldrich.

### Cell Culture

NCI-H446 and NCI-1688 (human SCLC) cell lines from our previous published paper [15] were cultured in RPMI-1640 supplemented with 10% HyClone fetal bovine serum (FBS) (ThermoFisher Scientific, Fremont, CA, USA) in an atmosphere of 5% CO_2_ at 37°C.Cells were grown in 75 cm^2^ culture flasks and harvested in a solution of trypsin-EDTA at the logarithmic growth phase.

### Plasmids and siRNAs

The plasmids pcDNA6.2-STAT3 and control vectors(a dominant-negative mutant of STAT3) were described previously [33]. siRNA sequences targeting STAT3α were as follows: F,5′-TGCTGAATGCAGGCAATCTGTTGCCGGTTTTGG CCACTGACTGACCGGCAACATTGCCTGCATT-3′ and R,5′-CCTGAATGCAGG CAATGTTGCCGGTCAGTCAGTGGCCAAAACCGGCAACAGATTGCCTGCATTC-3′. Negative control: F,5′-TGCTGAAATGTACTGCGCGTGGAGACGTTTTG GCCACTGACTGACGTCTCCACGCAGTACATTT-3′ and R, 5′-CCTGAAATGTA CTGCGTGGAGACGTCAGTCAGTGGCCAAAACGTCTCCACGCGCAGTACATTC-3.

### Transient and Stable Transfection

Transfection of plasmids and siRNAs into small cell lung cancer cells was done by using Lipofectamine 2000 (Invitrogen). For transient transfection, cells were transfected with siRNA or plasmids at different doses as indicated for 48 hours before functional assays were carried out. Stably transfeted cell lines were isolated from SCLC cells transfected with pcDNA6.2-STAT3 plasmid via selection with Blasticidin (Invitrogen, 5 µg/ml).

### Cell Proliferation Assay

Proliferation of SCLC cells was determined by analysing 5′-Bromo-2′-deoxyuridine (Brdu) incorporation into newly synthesised DNA using a cell proliferation enzyme linked immunosorbent assay (Cell proliferation ELISA (Brdu), Roche, Mannhein, Germany). For the Brdu incorporation assay, cells were counted and seeded into 96-well culture plates (2×10^3^ cells/well). Brdu (10 µl/well) was added SCLC cells were fixed and stained after 4 h according to the manufacturer’s instructions. Colorimetric analysis was performed with an ELISA plate reader (DTX880; Beckman, Miami, FL) at 450 nm.

### Cell Cycle Analysis by Flow Cytometry

After treatment with curcumin for 24 h, SCLC cells were harvested and washed twice with cold phosphate-buffered saline (PBS) and fixed in 75% ethanol for 2 h at 4°C. The fixed cells were washed twice with 500 ml of cold PBS. Cells then were stained with 500 ml of propidium iodide (PI) staining solution (50 mg/ml PI, 0.1%Triton X-100, 200 mg/ml DNase-free RNase in PBS) for 30 min at room temperature in the dark. Ten thousand events per sample were acquired using a FACS-scan flow cytometer (Becton-Dickinson, San Jose, CA, USA), and the percentage of cells in G_0_/G_1_, S, G_2_/M and Sub-G_2_/M phases of the cell cycle were determined using CELLQuest software (Becton-Dickinson).

### The Wound-Healing Assay

The SCLC cells were cultured in 24-well plates and grown in medium containing 10% FBS to nearly confluent cell monolayer, then carefully scratched using a plastic pipette tip to draw a linear “wound” in the cell monolayer of each well. The monolayer was washed twice with PBS to remove debris or the detached cells from the monolayer, and then curcumin was added by 15 µM; IL-6 was added by 25 ng/ml; the control well was added with 0.1% of DMSO as the solvent control. The cultures were incubated at 37°C and photographedimmediately and monitored by time lapse in the Carl Zeiss Axiovert Bio-station system. Under the microscope, the number of cells that migrated into the cell-free zone, base on the line of the linear “wound” was evaluated. The experiments were performed thrice in triplicate and were counted double blind by at least two investigators.To investigate cell motility, monolayers of SCLC cells were prepared and wounded as described above.The wounded monolayers were incubated at 37°C in RPMI-1640 containing 10% FBS with mitomycin C (10 µg/ml, Sigma) to block mitosis.

### The Transwell Migration Assay

The cancer cell transwell migration assay was performed according to previous study and partially modified [34]. Briefly, Transwell membrane (Corning Costar Corporation) was used. The SCLC cells were trypsinized, washed, and kept suspended in medium without FBS. To the lower wells of the chambers, migration-inducing medium (with 10% FBS) were added. Upper wells were filled with serum-free medium with cells (20,000 cells per well), in some cases, also containing 15 µmol/L of curcumin or 25 ng/ml of IL-6. Then, the chamber was placed into a humidified incubator. After 8 h, assays were stopped by removal of the medium from the upper wells and careful removal of the filters. Filters were fixed with methanol by brief submersion and were subsequently wiped on the cells on the upper side using the Q-tip. Filters were stained with 20% Giemsa solution for the light microscope or 4–6-diamidino-2-phenylindole for the fluorescent microscopy–based high content screening system (Cellomics). Evaluation of completed transmigration was performed under the microscope, and random fields were scanned (four fields per filter) for the presence of cells at the lower membrane side only.

**Table 2:** The primer sequences for RT-PCR.

Gene	Primer Sequences
STAT3	Forward 5’-CTGGCCTTTGGTGTTGAAAT-3’
STAT3	Reverse 5’-AAGGCACCCACAGAAACAAC-3’
Cyclin B1	Forward 5’-CAGTCAGACCAAAATACCTACTGGGT-3’
Cyclin B1	Reverse 5’-ACACCAACCAGCTGCAGCATCTTCTT-3’
GAPDH	Forward 5’-AGGTCGGAGTCAACGGATTTG-3’
GAPDH	Reverse 5’-GTGATGGCATGGACTGTGGT-3’

### Invasion Assay

The cancer cell invasive ability with or without indicated treatment was examined by membrane transwell culture system. Briefly, Transwell membrane (Corning Costar Corporation) coated with Matrigel (2.5 mg/ml; BD Biosciences Discovery Labware) was used for invasion assay. Cells were trypsinized, centrifuged, and resuspended at 10^6^ cells/ml in RPMI 1640. Cells were seeded onto the upper wells of precoated transwells, 2×10^4^ cells per well. Lower wells of the transwells contained the same medium with 10% FBS. After 16 h of incubation, the cells on the upper well and the membranes coated with Matrigel were swabbed with a Q-tip, fixed with methanol, and stained with 20% Giemsa solution. The cells that penetrated through filter were counted at a magnification of ×200 in 15 randomly selected fields, and the mean number of cells per field was recorded. The experiments were performed thrice in triplicate.

### Colony Formation in Soft-Agar Assay

Cells (5×10^3^ cells per well) were mixed with 0.3% agar solution in RPMI 1640 containing 10% FBS, and the solution was poured on top of a 0.60% agar layer containing RPMI 1640 and 10% FBS in 12-well tissue culture plates. After 15 days in culture, colonies were then stained with 0.005% crystal violet and examined using Bio-Rad Quantity One software (Hercules, CA).

### Reverse Transcription–Polymerase Chain Reaction Assay

Total RNA was isolated from cells using TRIzol (Invitrogen) according to the manufacturer’s instructions. To determine the efficiencies of siSTAT3 and control siRNA, semi-quantitative RT-PCR was performed on a G-STORM thermal cycler (GRI Ltd, Byfleet, UK) using the TaKaRa RNA PCR Kit (AMV) Ver.3.0 (TaKaRa, Dalian, China). The primers were designed and synthesized by Shenggong Biotech Company according to the serial number from GenBank as shown in Table 2. The reaction was started at 94°C for 2 min, amplified for 35 cycles of 30 s at 94°C, 60 s at 55°C (for STAT3), 45 s at 57°C (for CyclinB1), or 30 s at 72°C and ended with a 5 min extension at 72°C. GAPDH mRNA was used as an internal control and coamplified with STAT3 or Cyclin B1 mRNA. The products were visualized by electrophoresis on a 1.5% agarose gel, and the density of each band was analyzed using the Gel Image Analysis System (Tanon 2500R).

### Western Blot

Total protein from cells was extracted in lysis buffer (Pierce) and quantified using the BCA method. A total of 20 µg of protein was separated using 10%–15% SDS-PAGE and then electrophoretically transferred to a PVDF membrane (Millipore).The membrane was blocked with 5% nonfat milk in TBST solution and incubated at 4°C overnight with primary antibody in blocking solution. After washing three times with TBST, the membrane was incubated at room temperature for 1 h with horseradish peroxidase–conjugated secondary antibody diluted in TBST. Protein bands were visualized using enhanced chemiluminescence (Pierce) and detected using. BioImaging Systems (UVP). The relative protein levels were calculated based on GAPDH protein as a loading control.

### Statistical Analysis

SPSS 13.0 software was used for statistical analysis. Data were summarized as mean ± SD. Student’s t-tests were used to determine the significant differences between groups. Results were considered to be significant for *p*-values of <0.05.
